# Medial collateral ligament reconstruction graft isometry is effected by femoral position more than tibial position

**DOI:** 10.1007/s00167-020-06420-8

**Published:** 2021-01-17

**Authors:** Christoph Kittl, James Robinson, Michael J. Raschke, Arne Olbrich, Andre Frank, Johannes Glasbrenner, Elmar Herbst, Christoph Domnick, Mirco Herbort

**Affiliations:** 1 Department of Trauma, Hand and Reconstructive Surgery, Westphalian Wilhelms University Muenster, Muenster, Germany; 2 Avon Orthopaedic Centre, Bristol, UK; 3 Department of Trauma and Hand Surgery, Euregio Klinik Nordhorn, Nordhorn, Deutschland; 4 OCM Clinic, Munich, Germany

**Keywords:** Medial collateral ligament, Posterior oblique ligament, MCL reconstruction, POL reconstruction, Length change pattern, Isometry

## Abstract

**Purpose:**

The purpose of this study was to examine the length change patterns of the native medial structures of the knee and determine the effect on graft length change patterns for different tibial and femoral attachment points for previously described medial reconstructions.

**Methods:**

Eight cadaveric knee specimens were prepared by removing the skin and subcutaneous fat. The sartorius fascia was divided to allow clear identification of the medial ligamentous structures. Knees were then mounted in a custom-made rig and the quadriceps muscle and the iliotibial tract were loaded, using cables and hanging weights. Threads were mounted between tibial and femoral pins positioned in the anterior, middle, and posterior parts of the attachment sites of the native superficial medial collateral ligament (sMCL) and posterior oblique ligament (POL). Pins were also placed at the attachment sites relating to two commonly used medial reconstructions (Bosworth/Lind and LaPrade). Length changes between the tibiofemoral pin combinations were measured using a rotary encoder as the knee was flexed through an arc of 0–120°.

**Results:**

With knee flexion, the anterior fibres of the sMCL tightened (increased in length 7.4% ± 2.9%) whilst the posterior fibres slackened (decreased in length 8.3% ± 3.1%). All fibre regions of the POL displayed a uniform lengthening of approximately 25% between 0 and 120° knee flexion.

The most isometric tibiofemoral combination was between pins placed representing the middle fibres of the sMCL (Length change = 5.4% ± 2.1% with knee flexion). The simulated sMCL reconstruction that produced the least length change was the Lind/Bosworth reconstruction with the tibial attachment at the insertion of the semitendinosus and the femoral attachment in the posterior part of the native sMCL attachment side (5.4 ± 2.2%). This appeared more isometric than using the attachment positions described for the LaPrade reconstruction (10.0 ± 4.8%).

**Conclusion:**

The complex behaviour of the native MCL could not be imitated by a single point-to-point combination and surgeons should be aware that small changes in the femoral MCL graft attachment position will significantly effect graft length change patterns. Reconstructing the sMCL with a semitendinosus autograft, left attached distally to its tibial insertion, would appear to have a minimal effect on length change compared to detaching it and using the native tibial attachment site. A POL graft must always be tensioned near extension to avoid capturing the knee or graft failure.

## Introduction

The three principal structural elements of the “medial ligament complex (MCL)” have been described as the superficial MCL (sMCL), the deep MCL (dMCL) and the posterior oblique ligament (POL) [16, 37, 45].

There is a wide consensus that the majority of isolated grade I and II MCL injuries heal with rehabilitation alone [12, 18, 20]. Most patients return to sports at 3 months and there are excellent long-term patient-reported outcomes [29]. Although grade III injuries also may heal without surgery, some patients remain symptomatic following conservative treatment, necessitating reconstruction [20]. Combined sMCL and posterior oblique ligament (POL) injuries may be associated with an increased incidence of failure to respond to non-operative treatment and may result in persistent MCL instability [31].

Residual peripheral ligament laxity is an important cause of ACL graft laxity and failure [41]. The ACL functions in conjunction with the medial ligament complex to prevent anteromedial instability and is a secondary restraint to valgus rotation [48]. Thus, if the medial structures remain compromised, the ACL graft may be exposed to increased loads, potentially leading to graft failure [4, 5]. Similarly, although combined high-grade MCL and PCL injuries are rarer [19], because, particularly the POL has a role in restraining both posterior translation and internal rotation in extension [45], the need for combined medial and PCL reconstruction has been suggested [6, 18, 26, 35, 40].

Medial reconstructions are challenging as it is difficult to reproduce the biomechanical behaviour of flat, sheet-like structures [30, 39]. It is a widely accepted principle that isometric ligament reconstruction may be advantageous in reducing the likelihood of unwanted graft behaviour [1]. Inappropriate sMCL graft positions could result in abnormal graft tensioning patterns leading to either persistent laxity or over-constraint [2]. However, graft positioning is complicated by controversy about the location of the sMCL femoral attachment. Historically, this has been described as being on the medial epicondyle (ME) [8, 17, 24, 45], whereas more recently, LaPrade et al. [23] reported that the sMCL attaches to a depression 5 mm posterior and 3 mm proximal to the ME. In contrast, Liu et al. [28] have described the fibres enveloping the ME. These different interpretations of the anatomy may relate to the fact that there is a confluence of fibres in the region of the ME that makes it difficult to identify a precise attachment site.

Conversely, the native POL is known to be anisometric [41] restraining valgus, internal tibial rotation and posterior tibial translation near extension and slackening as the knee flexes. Little is known about the precise length change pattern of its fibres. It has a linear attachment extending from just posterior to the attachment of the longitudinal parallel fibres of the sMCL around the base of the adductor tubercle in a posterior and then slightly proximal direction. Reconstruction of the central arm of the POL has been suggested [9] but the length changes of a graft in this position are ill defined.

The goal of this study was to (1) examine the length change patterns of the native medial structures of the knee and (2) determine the effect on graft length change patterns for different tibial and femoral attachment points for previously described medial reconstructions. The aim was to recommend optimal femoral and tibial attachment positions for medial reconstructions that closely reproduce native ligament length change behaviour.

## Materials and methods

Eight fresh–frozen cadaveric knee specimens were obtained from the local tissue bank. The knee specimens were dissected and tested with the necessary permissions from the “Gesetz über das Leichen-, Bestattungs und Friedhofswesen (Bestattungsgesetz) des Landes Schleswig–Holstein vom 04.02.2005, Abschnitt II, § 9 (Leichenöffnung, anatomisch)”.

### Specimen preparation

The fresh–frozen specimens were thawed 24 h prior to testing. The femur and the tibia were cut approximately 150 mm above and below the joint line, respectively. The skin and subcutaneous fat were resected and all other structures were left intact. An intramedullary rod was cemented into the femoral/tibial shaft using polymethylmethacrylate (PMMA) and a screw acting as a locking bolt for rotational stability. Consistent with previous studies [14, 22, 42], the quadriceps muscle and the iliotibial tract was divided into six different anatomic parts: rectus femoris, vastus lateralis longus, vastus lateralis obliquus, vastus medialis longus, vastus medialis obliquus, and the iliotibial tract. Cloth strips were sutured to the musculotendinous junction of each muscle part to prevent slippage of the loading cables. On the medial side, the layer 1 fascia was divided and the proximal and distal attachment sites of the sMCL and the POL were carefully exposed.

**Fig. 1:**
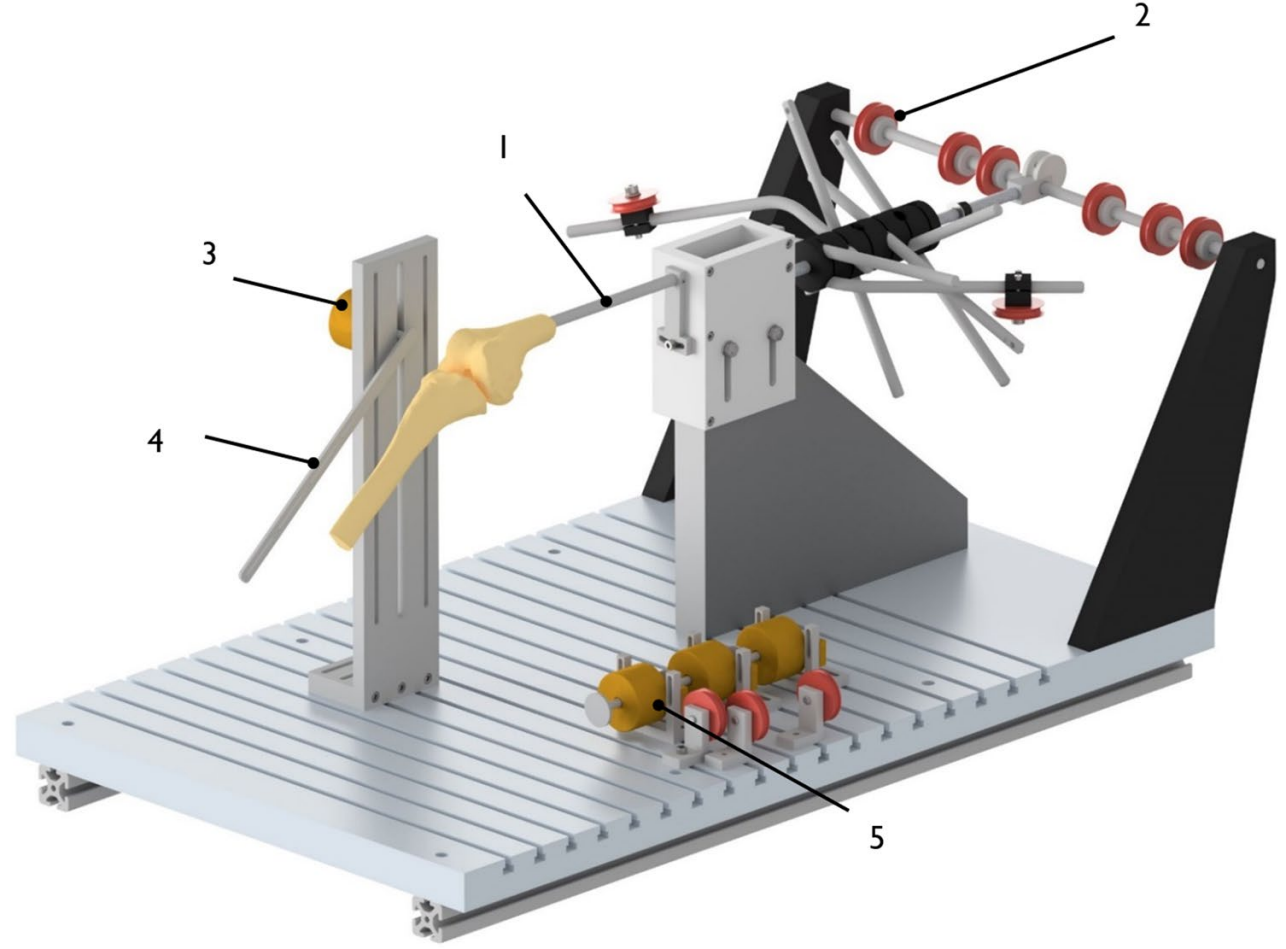
The knee was rigidly mounted into an open chain muscle extension rig using an intramedullary femoral rod (1). The quadriceps muscle parts and the iliotibial tract were loaded according to their cross-section area and their fibre orientation using a pulley system (red wheels; 2) and hanging weights. The tibia was free to rotate and could be manually flexed to 120°. A rotary encoder (3), which was attached to a metal bar (4) and secured to the tibia via a K-wire, recorded the knee flexion angle. A second rotary encoder (5) recorded the distance between two tibiofemoral points, using a monofilament suture. This rig was adapted and modified with rotary encoders from Ghosh et al. [15]

After the preparation, the specimen was placed parallel to its posterior condylar axis into a custom-made rig (Fig. 1), which has been previously shown to have a high test–retest reliability [42]. The femoral intramedullary rod was secured via a connection rod and two clamps to prevent rotation. The quadriceps muscle parts and the ITB were then loaded using hanging weights (total of 205 N) according to their cross-section areas and their fibre orientation via a pulley system. This quadriceps tension extended the knee, which could then be manually flexed from 0 to 120º continuously.

**Fig. 2:**
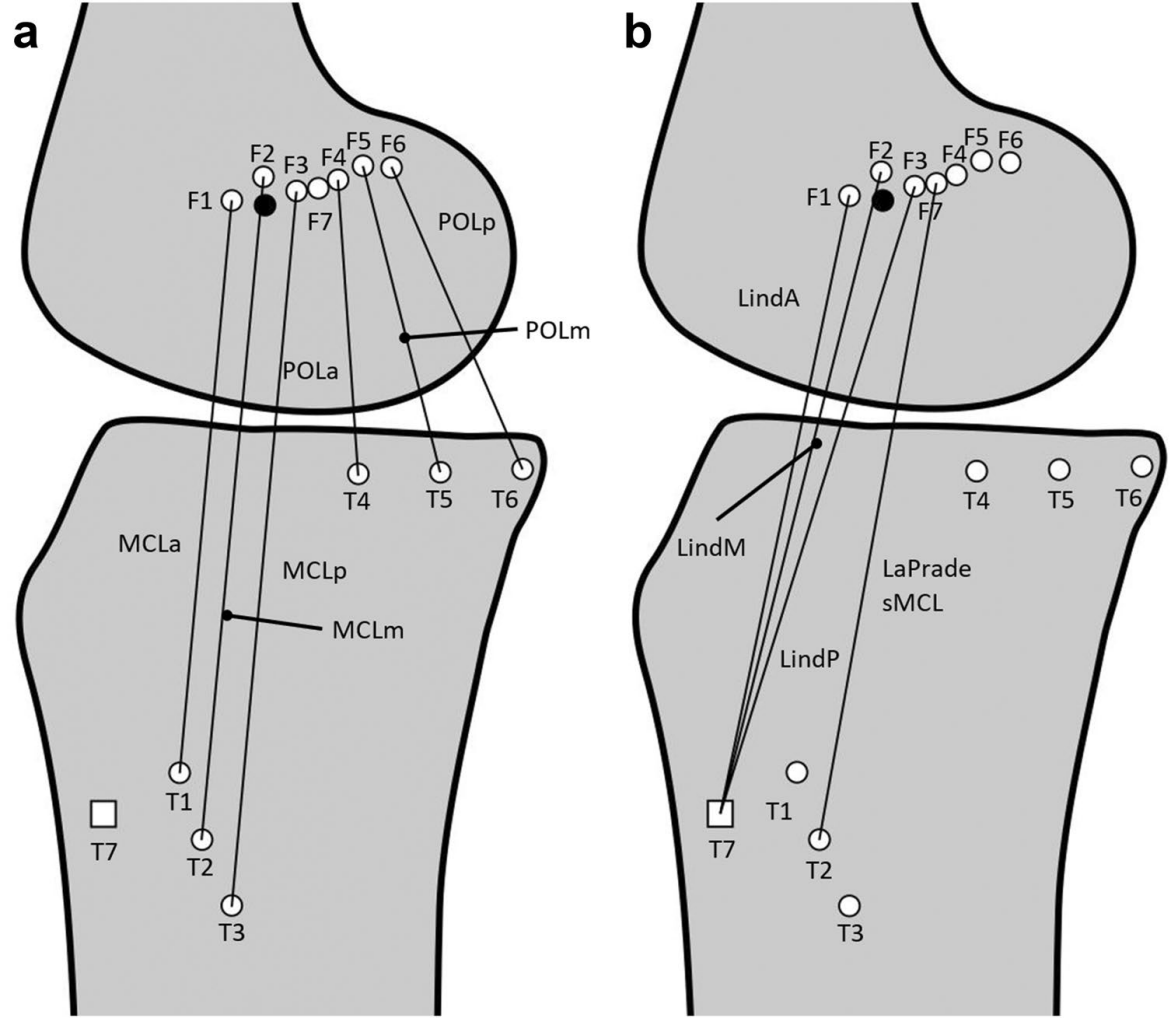
**a** Position of tibial pins and femoral eyelets corresponding to the native fibres of the sMCL and the posterior oblique ligament (POL) and **b** the positions of sMCL and POL reconstructions. Black dot: medial epicondyle (ME); square: semitendinosus insertion site

Different tibial and femoral attachment positions were marked by small pins (Fig. 2; Table 1).

**Table 1:** Position of the femoral and tibial pin and the corresponding tibiofemoral combination

Tibiofemoral combination	Position of the femoral pin	Position of the tibial pin
*Native medial structures*		
Anterior fibres of the MCL (T1–F1)	Anterior edge of the ME	Anterior tibial MCL attachment
Middle fibres of the MCL (T2–F2)	Superior edge of the ME	Middle distal tibial MCL attachment
Posterior fibres of the MCL (T3–F3)	Posterior edge of the ME	Posterior distal tibial MCL attachment
Anterior fibres of the POL (T4–F4)	Anterior edge of the femoral POL attachment	Anterior edge of the tibial POL attachment
Middle fibres of the POL (T5–F5)	Middle portion of the femoral POL attachment	Middle portion of the tibial POL attachment
Posterior fibres of the POL (T6–F6)	Posterior edge of the femoral POL attachment	Posterior edge of the tibial POL attachment
*Medial reconstructions*		
Anterior Lind/Bosworth reconstruction (T7–F1)	Anterior edge of the ME	Semitendinosus insertion
Middle Lind/Bosworth reconstruction (T7–F2)	Proximal edge of the ME	Semitendinosus insertion
Posterior Lind/Bosworth reconstruction (T7–F3)	Posterior edge of the ME	Semitendinosus insertion
Anatomic sMCL reconstruction described by LaPrade et al. (T2–F7)	5 mm posterior and 3 mm proximal to the tip of the ME	Middle distal tibial MCL attachment

*ME* medial epicondyle

The most anterior fibres of the native sMCL were traced from their tibial attachment from distal to proximal. A pin was placed at the tibial attachment distally and another pin to the femoral attachment. The most posterior sMCL fibres were marked in a similar manner. The middle fibres were defined as being equidistant between the anterior and posterior fibres, and their tibial and femoral attachments were traced and marked with pins. Proximally, the central fibres of the sMCL were found to attach just proximal to the ME as described by the previous authors [28]. For the POL, the anterior-most fibres of the superficial arm (the thin fascial expansion at the posterior border of the sMCL [37]) were traced between tibial and femoral attachments which were then marked with pins. The attachments of central arm of the POL was similarly marked as were the posterior fibres of the POL (the capsular arm). In some smaller knees (*n* = 5), the posterior MCL and the anterior POL femoral attachment overlapped, so that only one pin was used for both attachment points.

To assess length change patterns of medial reconstruction grafts, additional pins were placed at the graft attachment positions described in the surgical techniques. The modified Bosworth reconstruction described by Lind et al. [26] utilises a semitendinosus autograft left attached to its tibial insertion, and stripped proximally. A pin was placed in the insertion of the semitendinosus at the pes anserinus (position T7). The semitendinosus tendon is re-routed proximally to a femoral tunnel to reconstruct the sMCL and taken back distally to the tibia to reproduce the POL. The reconstruction technique describes adjusting the position of the femoral tunnel to best achieve isometric graft behaviour. Length changes of this reconstruction with three pin positions in the anterior, middle and posterior parts of the femoral attachment (F1, F2 and F3) were tested. LaPrade et al. [23] recommended that the femoral tunnel for sMCL reconstruction is placed 3 mm proximal and 5 mm posterior to the ME. This position (F7) was accordingly marked in all specimens. Distances from the ME were measured to the nearest 0.1 mm using a digital calliper (accuracy ± 0.01 mm).

Length changes were then measured using a braided high-resistant suture tied to a tibial pin, passed around a femoral pin (corresponding to fibre/reconstruction being investigated) and led to an optical rotary incremental encoder (Opkon, PRID 58H8, Istanbul, Turkey) via a pulley. A 100-mm circumference custom-made rubber-edged measuring wheel was attached to the rotating part of the rotary encoder. Friction between the suture and rubber-edged wheel resulted in rotation. The suture was always held taught using a small weight (0.3 N). The accuracy of the optical rotary encoder was  ± 0.08°, allowing length changes to be calculated to the nearest 0.1 mm (accuracy ± 0.02 mm).

A K-wire (2.4 mm) was then drilled through the tibia and attached to a metal bar (see 4 in Fig. 1), which could measure the angle of flexion using a rotary encoder with its centre positioned on a metal stand aligned to the axis of flexion/extension.

Each tibiofemoral combination was tested three times. The signals of the rotatory encoders were then recorded using a microcontroller and a computer interface. These signals were collected for each tested tibiofemoral combination and converted into length changes (mm; rotary encoder 1) and knee flexion angle (°; rotary encoder 2) using a custom-made tool. A MATLAB script was designed to average the three cycles in increments of 0.5°.

### Data analyses

Length change pattern of the native structures and the reconstructions were then plotted. To calculate the strain, [(length change/absolute length at 0°) × 100%] the absolute length of each tested tibiofemoral attachment combination was measured at full extension using a digital calliper with an accuracy of 0.01 mm. The level of isometry was displayed using the TSR, which was calculated subtracting the minimum strain from the maximum strain for each combination. High values of TSR reflect non-isometry, whereas low values of TSR display near isometry.

### Statistical analysis

Statistical analysis was performed using SPSS v21 (Statistical Package for the Social Sciences, IBM Corp., Armonk, New York, US). For each tibial/femoral attachment combination tested, a datapoint each 10° as the knee was flexed from 0° to 120° was taken. Two three-way repeated-measures ANOVAs were conducted to determine the effect of changing the femoral or tibial insertion site. Multiple two-way ANOVAs were conducted to compare length changes and TSR for the native fibres and reconstructions. Significance was set at *p* < 0.05 divided by the number of tests (Bonferroni correction).

Based on previous work [22, 50], an a priori power analysis was performed to detect a difference of 1% strain (Effect size 0.78; Power 0.8) and 3% total strain range (TSR, Effect size 0.73; Power 0.8) using G*Power (Universität Düsseldorf, Germany). For this, an estimated total sample size of six was calculated.

## Results

### Native sMCL

**Fig. 3:**
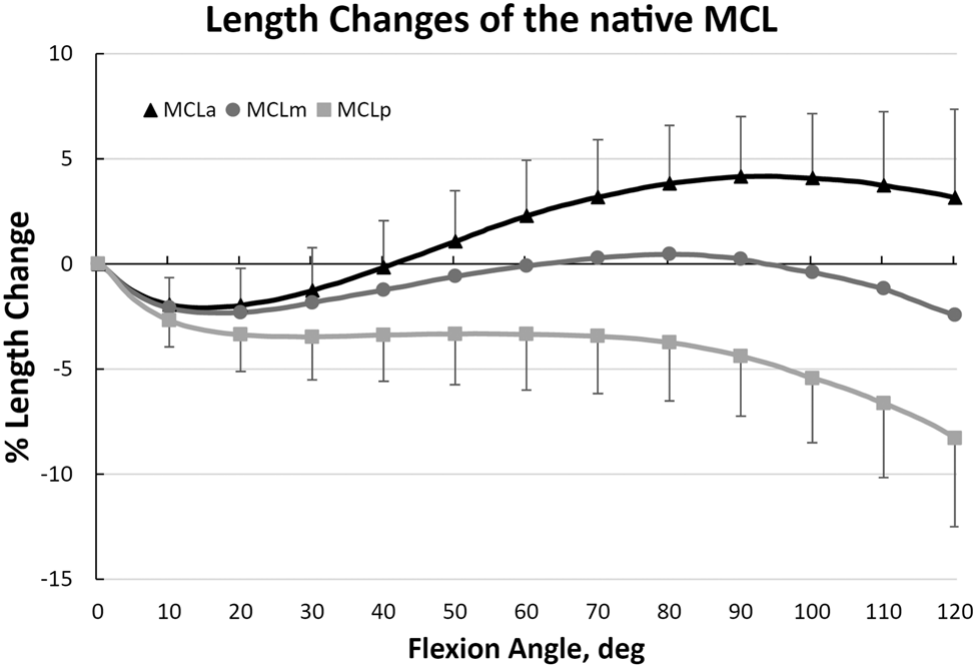
Length change pattern of the different fibre regions of the native sMCL with a pooled 95% confidence interval. The anterior fibre region was tight in flexion, whereas the posterior fibre region was tight in extension

The sMCL fibres demonstrated different length change patterns with knee flexion. The anterior fibres tightened in flexion, whereas the posterior fibres were tighter in extension (*p* < 0.05). The anterior fibres showed a constant increase in length from 20° to 70° (5.2%) knee flexion (Table 1), which was followed by a quasi-isometric area from 70 to 120° knee flexion (Fig. 3). Conversely, the posterior fibre region first presented an isometric region at early flexion angles (20–80°), followed by a decrease of length between 80 and 120° knee flexion (− 3.7 ± 1.5% to 8.3 ± 3.1%). The middle fibre region of the MCL showed a sine wave behaviour, which presented an initial slackening from 0 to 20° knee flexion (− 2.3 ± 1.3%), similar to the anterior region. This was followed by a constant tightening towards 80° of knee flexion (0.5 ± 2.7%) and a decrease of length at late knee flexion (− 2.4 ± 4.2%).

### Native POL

**Fig. 4:**
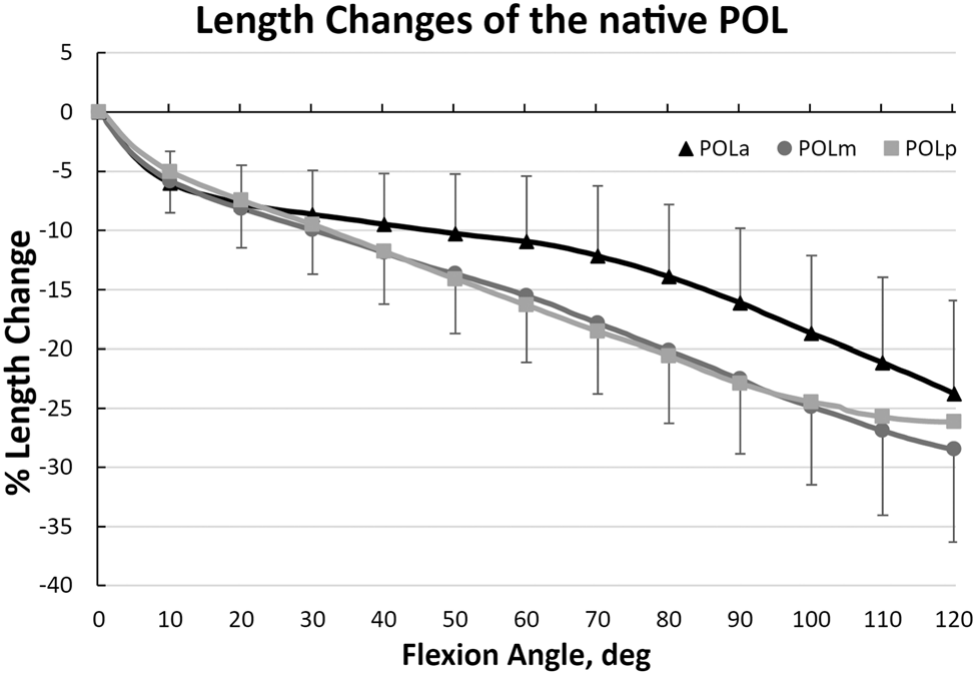
Length change pattern of the different fibre regions of the native POL with pooled 95% confidence interval. All three fibre regions showed a uniform decrease in length with knee flexion, which was not significant (n.s.)

All three fibre regions of the native POL presented a constant slackening towards 120° knee flexion (23.8–28.5%), which did not show a statistically significant length change pattern (n.s.; Fig. 4).

### Reconstructions

**Fig. 5:**
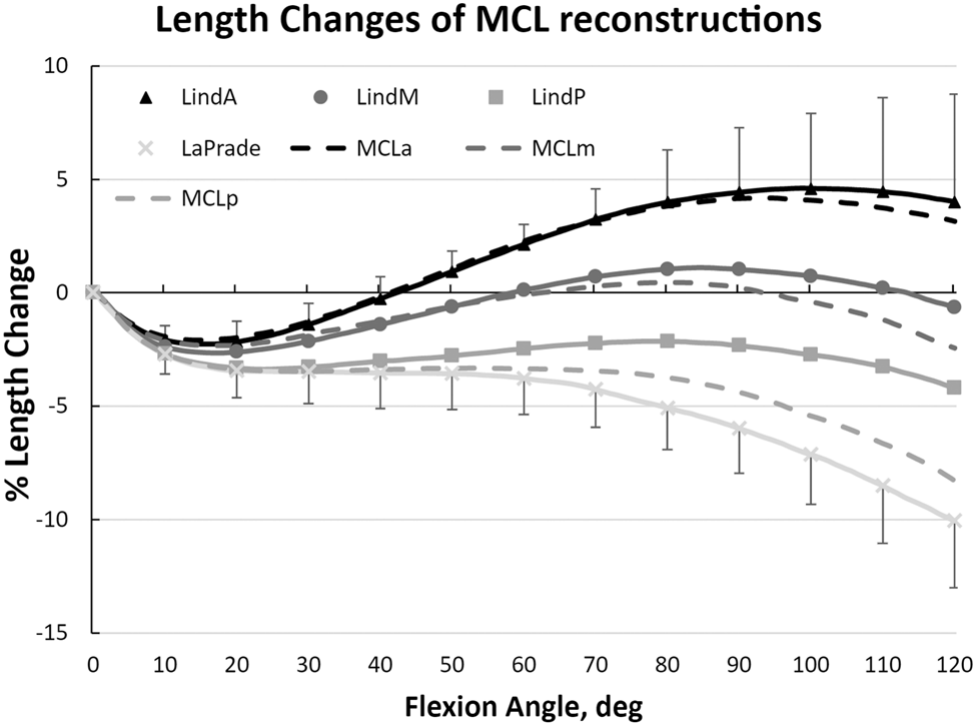
Length change patterns of the anterior (LindA, triangle), middle (LindM, circle), posterior (LindP, square), modified Bosworth reconstruction described by Lind et al. and the sMCL reconstruction according to the anatomical description of LaPrade et al. (LaPrade) with pooled 95% confidence interval. Length changes of the Lind and LaPrade reconstructions were not significantly different to the corresponding length changes of the native sMCL fibre regions (dashed lines)

The modified Bosworth reconstruction described by Lind et al. showed a similar length change pattern to that of the native MCL (Fig. 5). Compared to the length change patterns seen in the native sMCL, changing the tibial attachment to the insertion point of the semitendinosus appeared to have little effect on length change pattern (n.s.). Similar to the native sMCL fibre, the Lind reconstruction with an anterior femoral attachment (F1) was tighter in flexion, whereas with a more posterior femoral attachment (F3) was tighter in extension (*p* < 0.001).

The LaPrade sMCL reconstruction (T2–F7) demonstrated a significant decrease in length (i.e. slackening with progressive knee flexion) compared to the anterior native sMCL fibres (*p* < 0.001).

### Total strain range

The most isometric tibiofemoral combination of the native structures on the medial side was the middle portion of the MCL, which had a TSR of 5.4 ± 2.1% (Table 2). Conversely, the native POL presented the most non-isometric length change pattern (TSR of 23.8 ± 8% to 28.6 ± 6%).

**Table 2:** Total strain range of each tested tibiofemoral combination

Tibiofemoral combination	Total strain range
*Native media structures*	
Anterior fibres of the SMCL (T1–F1)	7.4 ± 2.9%
Middle fibres of the MCL (T2–F2)	5.4 ± 2.1%
Posterior fibres of the MCL (T3–F3)	8.3 ± 3.1%
Anterior fibres of the POL (T4–F4)	23.8 ± 8.0%
Middle fibres of the POL (T5–F5)	28.6 ± 6.0%
Posterior fibres of the POL (T6–F6)	26.3 ± 9.2%
*Medial reconstructions*	
Anterior Lind/Bosworth reconstruction (T7–F1)	7.4 ± 2.7%
Middle Lind/Bosworth reconstruction (T7–F2)	5.6 ± 1.5%
Posterior Lind/Bosworth reconstruction (T7–F3)	5.4 ± 2.2%
Reconstruction of the MCL according to the anatomical description of LaPrade et al. (T2–F7)	10.0 ± 4.8%

Native medial structures

The most isometric Bosworth/Lind reconstruction was when the femoral attachment was placed at F2 (middle; TSR = 5.6 ± 1.5) and F3 (posterior; TSR = 5.4 ± 2.2).

## Discussion

The most important findings of this study were that small changes in the femoral attachment site of medial reconstructions resulted in significant changes in fibre length change behaviour and that utilising the tibial insertion of the semitendinosus tendon (as described for a Bosworth/Lind reconstruction) had no significant effect on fibre length change pattern.

The length change patterns of the native MCL strongly depended on its fibre region. The anterior part of the MCL was tight in flexion, whereas the posterior part of the MCL was tight in extension (*p* < 0.001), implying a reciprocal tensioning pattern of these fibre regions. This reciprocal behaviour of the MCL was first observed by Brantigan and Voshell [7] and later confirmed by several other works [3, 13, 21, 33, 46]. Recently, Willinger et al. [50] looked at the length change pattern of the anterior and posterior borders of the sMCL of 10 cadaveric knee specimen using a kinematics rig. The anterior region of the MCL showed a lengthening of 6% at 0–100° knee flexion and the posterior portion presented a 6% decrease of length at 100° knee flexion. This is similar to the present study’s lengthening of 4.1 ± 3.7% until 100° of flexion and a decrease of length for the posterior border of 5.4 ± 1.9% at 100° knee flexion. However, their increase and decrease of length was constant, whereas the present study showed an initial slackening of 2–3% (10–20° knee flexion) for all measured tibiofemoral MCL combinations. This initial slackening may be due to the external rotation of the tibia (screw home mechanism) in full extension, which will increase the length of the medial structures.

Several other studies [10, 11, 27, 43, 44, 47] found a constant decrease in length [11, 27] or a near-isometric behaviour [43] for the MCL, which was different to the present study. This is most likely due to differences in the testing setup, muscle loading, and dissection methods. Feeley et al. [11] assessed length changes of a modified Bosworth MCL reconstruction technique similar to the Lind reconstruction. They found a small, but significant difference in overall length change (2.7 ± 1.2 mm vs. 4.1 ± 2.3 mm) when the tibial attachment of the reconstruction was changed from the position of the proximal part of the sMCL tibial attachment site to the insertion to the semitendinosus The present study, however, did not find a significant difference, neither in TSR (5.4 ± 2.1 vs. 5.6 ± 1.5; n.s.), nor in overall length change pattern (n.s.).

It is known from previous studies that there is no perfect isometric tibiofemoral combination [38]. The MCL is an approximately 10-mm wide, flat structures with different fibre regions tensioning throughout flexion. Reconstructions of the medial side of the knee, however, cannot precisely imitate this complex tensioning pattern [49] and small changes in femoral graft position can result into different graft length change patterns. Moving the attachment point from the posterior edge of the ME to anterior will result in graft tightening rather than slackening in flexion. When performing medial reconstructions, surgeons should be aware of this effect when positioning the femoral tunnel to avoid unwanted graft behaviour as inappropriate graft tension can also induce over-constraint or ‘stretching out’ of the graft. If in doubt, an intra-operative check of the isometry should be performed.

The present study showed that there is an increase in the distance between femoral and tibial attachments of the POL of almost 30% as the knee flexes. The findings confirm that a POL graft should be tensioned and fixed with then knee in extension. Tensioning and fixation at higher knee flexion angles would lead to loss of motion and possibly graft failure due to rapid graft elongation likely to overcome the strain required for ligament failure [34].

This study had some limitations in addition those inherent in cadaveric knee biomechanical testing, including the age of specimens and number of knees tested. There are inconsistencies in the literature regarding the exact femoral attachment site of the sMCL. Some authors describe it posterior and proximal to the ME [11, 23], while others describe the sMCL as enveloping the ME [28, 36]. In this study, the knee was carefully dissected and the proximal attachment of the sMCL was found to envelope the ME. The anterior-most sMCL fibres attached to the anterior border of the ME, the posterior most fibres attached to the posterior border and the middle fibres to the superior border. However, it was also noted that in flexion the fibres of the sMCL appeared to bend posteriorly towards their femoral attachment as they coursed proximally from the tibia. This complex fibre pattern has been noted by the other authors [13, 32] and cannot be reproduced with sutures, which have a completely linear course, possibly leading to subtle length change differences for the native sMCL. However, the linear fibre course is representative of sMCL reconstruction.

In addition, fibre length change pattern in response to tibia-femoral rotatory loads (e.g. intern/external rotation, valgus rotation) were not investigated. Knees were also tested in an open chain extension rig against gravity, which only involved one muscle loading state. Different muscle loading states or involvement of the hamstring muscles were not considered. Finally, the present study setup only allowed for the measurement of length changes and not the measurement of actual tensile strain. This could be important further information for helping to determine the optimum angle of knee flexion angle for graft fixation and the amount of pre-tensioning required [25].

## Conclusion

The complex behaviour of the native MCL could not be imitated by a single point-to-point combination and surgeons should be aware that small changes in the femoral MCL graft attachment position will significantly effect graft length change patterns. Reconstructing the sMCL with a semitendinosus autograft, left attached distally to its tibial insertion, would appear to have a minimal effect on length change compared to detaching it and using the native tibial attachment site, perhaps obviating the need for tibial fixation. A POL graft should always be tensioned near extension to avoid capturing the knee or graft failure.
